# The Association of AMPK with ULK1 Regulates Autophagy

**DOI:** 10.1371/journal.pone.0015394

**Published:** 2010-11-03

**Authors:** Jong Woo Lee, Sungman Park, Yoshinori Takahashi, Hong-Gang Wang

**Affiliations:** Department of Pharmacology and Penn State Hershey Cancer Institute, The Pennsylvania State University College of Medicine, Hershey, Pennsylvania, United States of America; Wayne State University School of Medicine, United States of America

## Abstract

Autophagy is a highly orchestrated intracellular bulk degradation process that is activated by various environmental stresses. The serine/threonine kinase ULK1, like its yeast homologue Atg1, is a key initiator of autophagy that is negatively regulated by the mTOR kinase. However, the molecular mechanism that controls the inhibitory effect of mTOR on ULK1-mediated autophagy is not fully understood. Here we identified AMPK, a central energy sensor, as a new ULK1-binding partner. We found that AMPK binds to the PS domain of ULK1 and this interaction is required for ULK1-mediated autophagy. Interestingly, activation of AMPK by AICAR induces 14-3-3 binding to the AMPK-ULK1-mTORC1 complex, which coincides with raptor Ser792 phosphorylation and mTOR inactivation. Consistently, AICAR induces autophagy in TSC2-deficient cells expressing wild-type raptor but not the mutant raptor that lacks the AMPK phosphorylation sites (Ser722 and Ser792). Taken together, these results suggest that AMPK association with ULK1 plays an important role in autophagy induction, at least in part, by phosphorylation of raptor to lift the inhibitory effect of mTOR on the ULK1 autophagic complex.

## Introduction

Autophagy is an intracellular catabolic system that delivers cytoplasmic contents (e.g., proteins, lipids, and organelles) to lysosomes for degradation [1]. There are three major pathways of autophagy in eukaryotic cells, namely macroautophagy, microautophagy, and chaperone-mediated autophagy. Macroautophagy (referred to as autophagy hereafter) involves de novo formation of double-membrane vesicles, termed autophagosomes, which engulf intact organelles (such as mitochondria) and portions of the cytosol. The outer membrane of the autophagosome fuses with an endosome and/or lysosome to form an autolysosome where cytoplasm-derived materials, together with the inner membrane of the autophagosome, are degraded by lysosomal hydrolases. The resulting macromolecules are transported back to the cytosol as an internal source of nutrients to support energy production or biosynthesis. This self-digestion process is well conserved from yeast to human and has an impact on development, immune defense, programmed cell death, neurodegeneration, cancer, and aging [1], [2].

Target of rapamycin (TOR) is a serine/threonine protein kinase involved in regulating cell growth and metabolism [3]. Accumulating evidence indicates that TOR, as well as its mammalian homologue mTOR, functions as the major inhibitory controller of autophagy in the presence of growth factors and abundant nutrients [4]. Rapamycin, an inhibitor of TOR can induce autophagy even under nutrient rich conditions. In mammalian cells, mTOR is part of two structurally and functionally distinct multiprotein complexes termed mTORC1 (containing mTOR, GβL, PRAS40, and raptor), which is highly sensitive to rapamycin and cellular nutrient availability, and mTORC2 (containing mTOR, GβL, mSin1, and rictor), which is insensitive to rapamycin [3], [5]. Raptor is an essential protein for mTOR signaling that acts positively in mTORC1 as scaffold for recruiting downstream substrates such as ribosomal S6 kinase (p70S6K1) and eukaryotic initiation factor 4E binding protein 1 (4EBP1) to the mTORC1 complex. While nutrient-rich conditions activate mTORC1 through the small GTPase Rheb, nutrient-poor conditions inactivate mTORC1 through AMP-activated protein kinase (AMPK).

AMPK is a central metabolic sensor found in all eukaryotes that governs glucose and lipid metabolism in response to alterations in nutrients and intracellular energy levels. This serine/threonine kinase, which is a heterotrimer composed of a catalytic (AMPKα) subunit and two regulatory (AMPKβ and AMPKγ) subunits, is activated through the upstream kinase LKB1 when intracellular ATP levels decline and AMP levels increase, such as during nutrient and energy depletion or hypoxia [6]. Active AMPK directly phosphorylates the TSC2 tumor suppressor, leading to the inactivation of Rheb, which directly binds to and activates the mTORC1 kinase [7], [8]. Alternatively, a recent study indicated that AMPK regulates mTOR signaling by directly phosphorylating raptor on two conserved serine residues Ser722 and Ser792 [9]. The phosphorylation of raptor by AMPK induces 14-3-3 binding to raptor, which is required for the inhibition of mTORC1 and cell cycle arrest induced by energy stress [9].

The serine/threonine kinase Atg1 was the first autophagy regulator identified by genetic screens in yeast for essential autophagy genes [10]. Atg1 interacts with the autophagy regulatory proteins Atg13 and Atg17, and this complex is thought to function downstream of TOR [11], [12]. Atg13 is hyperphosphorylated in a TOR-dependent manner under nutrient-rich conditions and is rapidly dephosphorylated after nutrient starvation or rapamycin treatment, which enhances its interaction with Atg1. Both Atg13 and Atg17 are required for Atg1 kinase activity, autophagy induction, and cytoplasm-to-vacuole targeting [12]. In *Drosophila*, Atg1 physically interacts with TOR and Atg13, and both Atg1 and Atg13 are phosphorylated in a TOR- and Atg1-dependent manner under nutrient-rich conditions [13]. In contrast to yeast, *Drosophila* Atg13 is hyperphosphorylated under autophagic conditions, primarily via an Atg1-dependent pathway [13]. In mammals, there are two Atg1 homologues, UNC-51-like kinase 1 and 2 (ULK1 and ULK2). While ULK1 plays an important role in the regulation of autophagy, the function of ULK2 in autophagy has not been clearly defined. Similarly to yeast Atg1, ULK1 forms a complex with mammalian Atg13 (mAtg13) and FIP200, the functional analog of yeast Atg17 [14], [15], [16]. In this complex, mAtg13 mediates the interaction between FIP200 and ULK1 and is required for the phosphorylation of FIP200 by ULK1 in response to starvation [15]. Moreover, FIP200 is required for the stability and full kinase activity of ULK1 [14], [16].

Interestingly, it has recently been shown that mTORC1 associates with the ULK1-mAtg13-FIP200 complex through an interaction between raptor and ULK1 and phosphorylates ULK1 and mAtg13 to repress the kinase activity of ULK1, thereby suppressing the initiation of autophagy [15], [16], [17]. However, the mechanism by which mTOR is controlled to activate ULK1 activity for autophagy induction has not yet been clearly elucidated.

In this study, we have identified AMPK as a new ULK1-binding protein and demonstrated that raptor phosphorylation by AMPK plays an important role in autophagy induction, presumably through the suppression of the inhibitory effect of mTORC1 on the ULK1 autophagic complex under nutrient or energy stress conditions.

## Results

### Identification of AMPK as an ULK1-binding protein

**Figure 1:**
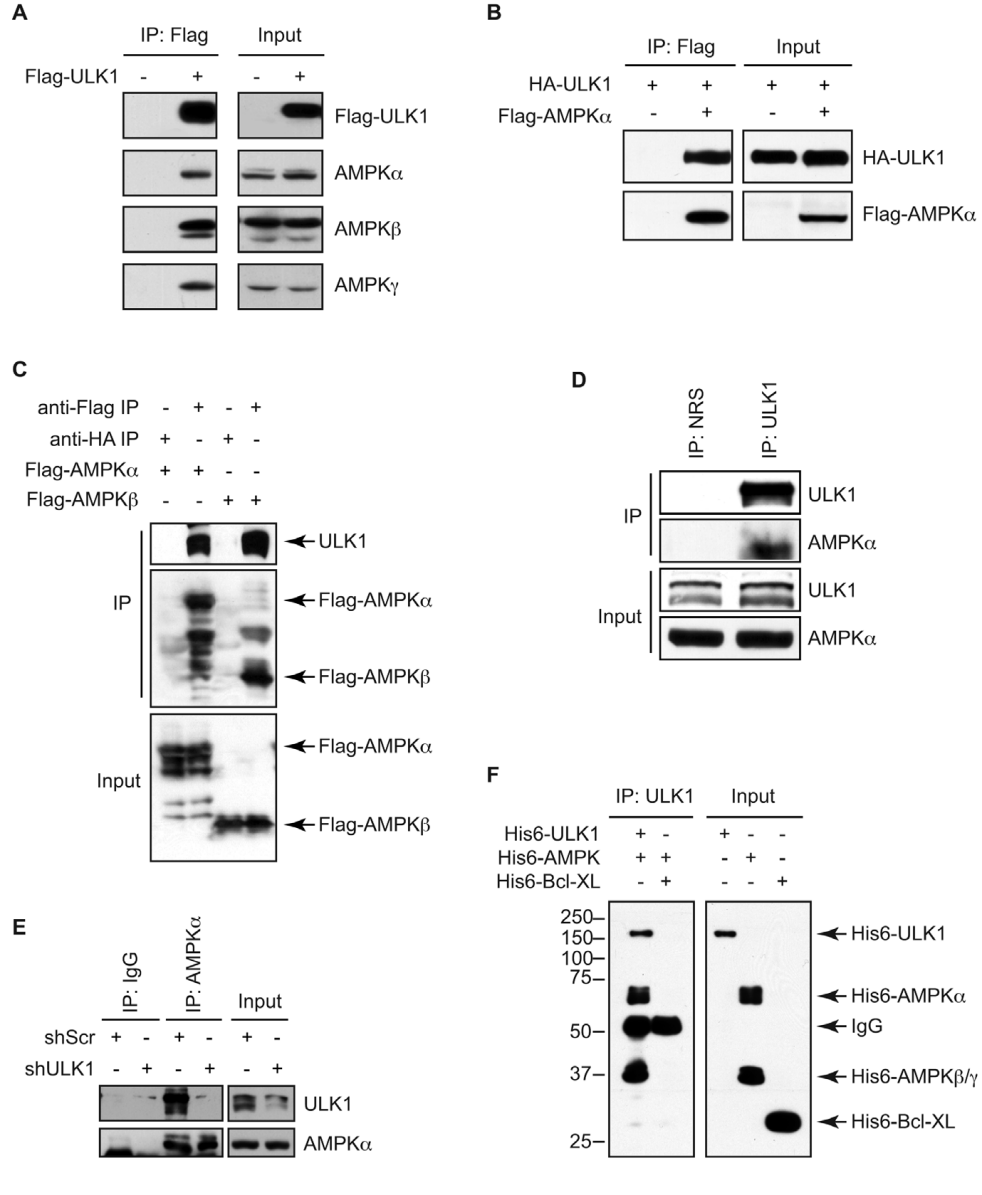
AMPK interacts with ULK1 directly. (A) 293T cells were transfected with Flag-ULK1 or control empty vector and subjected to immunoprecipitation with anti-Flag antibody, followed by immunoblot analysis with the indicated antibodies. (B) 293T cells were co-transfected with Flag-AMPKα2 and HA-ULK1. Cell lysates were subjected to immunoprecipitation using anti-Flag antibody followed by SDS-PAGE/immunoblot analysis with anti-HA and anti-Flag antibodies. (C) 293T cells were transiently transfected with Flag-AMPKα2 or Flag-AMPKβ1. After 24 h, immunoprecipitation was performed using anti-Flag or control anti-HA antibodies and analyzed by immunoblotting with anti-ULK1 and anti-Flag polyclonal antibodies. (D) 293T cell lysates were subjected to immunoprecipitation with anti-ULK1 polyclonal antibody or control preimmune rabbit serum (NRS), followed by immunoblot analysis with anti-ULK1 and anti-AMPKα antibodies. (E) 293T cells were infected with ULK1 shRNA or control shRNA lentiviruses and subjected to immunoprecipitation with control rabbit IgG or anti-AMPKα antibody followed by immunoblotting with anti-ULK1 and anti-AMPKα antibodies. (F) Purified His6-AMPKα1/β1/γ1 fusion proteins (250 ng) were mixed with purified His6-tagged ULK1 (1 µg) or Bcl-XL (200 ng) proteins in 1% Triton X-100 lysis buffer containing protease inhibitors and subjected to immunoprecipitation with anti-ULK1 antibody. The resulting protein complexes and 10% of the input proteins were analyzed by immunoblotting with anti-His-Tag polyclonal antibody.

To date, several ULK1-interacting proteins have been reported in mammals, including GABARAP, GATE-16, SynGAP, Syntenin, mAtg13, FIP200, Atg101, p62/SQSTM1, and mTORC1 [14], [15], [16], [17], [18], [19], [20], [21]. To gain further insight into the mechanism of action of ULK1, we took a proteomics approach combined with tandem affinity purification (TAP) [22] to identify additional ULK1-binding protein(s) in 293 cells stably transfected with TAP-ULK1. Tandem mass spectrometry (MS/MS) analysis of the ULK1 immune complexes revealed AMPKγ1, an AMPK subunit, as a new ULK1-binding protein (data not shown). AMPK is a heterotrimeric complex composed of a catalytic subunit (AMPKα) and two regulatory subunits (AMPKβ and AMPKγ), and each subunit exists as different isoforms (α1, α2, β1, β2, γ1, γ2, γ3). During preparation of this manuscript for publication, Behrends et al [23] reported a detection of the catalytic and regulatory subunits of AMPK in ULK1 as well as ULK2 immunocomplexes by proteomic analysis. Consistently, all three endogenous AMPK subunits (AMPKα, AMPKβ, and AMPKγ) were confirmed to associate with HA-ULK1 in 293 cells by co-immunoprecipitation analysis (Fig. 1A). The interaction between AMPK and ULK1 was further confirmed in 293T cells co-transfected with Flag-AMPKα2 and HA-ULK1 (Fig. 1B). We also observed an association between endogenous ULK1 and Flag-tagged AMPKα2 or AMPKβ1 (Fig 1C). Moreover, endogenous ULK1 was co-immunoprecipitated with endogenous AMPKα in 293T cells (Fig 1D). This interaction is specific, as knockdown of ULK1 abrogated co-immunoprecipitation of ULK1 with AMPK (Fig 1E). In addition, purified recombinant His6-tagged AMPKα/β/γ subunits could form a complex with purified recombinant His6-ULK1 but not His6-Bcl-XL proteins *in vitro*, suggesting that the interaction between AMPK and ULK1 is a direct and specific association (Fig. 1F). Taken together, these data indicate that AMPK is a new binding partner for ULK1.

### AMPK binds to the PS domain of ULK1

**Figure 2:**
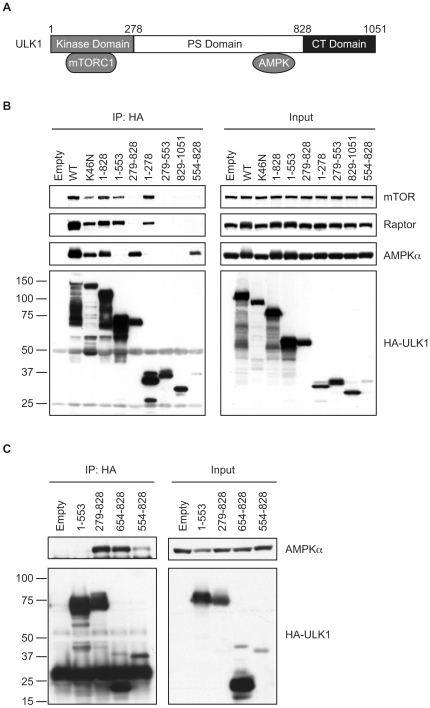
AMPK and mTORC1 interact with ULK1 through the PS domain and the kinase domain of ULK1, respectively. (A) A schematic of ULK1 illustrating its kinase domain, proline/serine-rich domain, C-terminal (CT) domain, and regions for binding to AMPK and mTORC1. (B, C) 293T cells were transfected with empty vector or HA-tagged wild type (WT) or deletion mutants of ULK1 and subjected to immunoprecipitation with anti-HA antibody. The resulting immune complexes were analyzed by immunoblotting with antibodies specific for mTOR, raptor, AMPKα or HA-tag.

To determine which region of ULK1 is required for its interaction with AMPK, we generated a series of ULK1 deletion mutants. Co-immunoprecipitation analyses revealed that AMPK binds to amino acids 654–828 within the proline-serine rich (PS) domain of ULK1 (Fig. 2A–C). However, in contrast to the previous report [17], the mTORC1-binding site was mapped to the kinase domain (amino acids 1–278) of ULK1 (Fig. 2A, B). Moreover, AMPK, Raptor and mTOR could interact with both wild type and kinase-dead mutant (K46N) of ULK1 (Fig. 2B), suggesting that the kinase activity is not required for ULK1 to interact with AMPK and mTORC1. Taken together, our results indicate that AMPK binds to the PS domain of ULK1, whereas mTORC1 associates with ULK1 through raptor binding to the kinase domain of ULK1.

### The AMPK binding domain is required for ULK1 to induce autophagy

**Figure 3:**
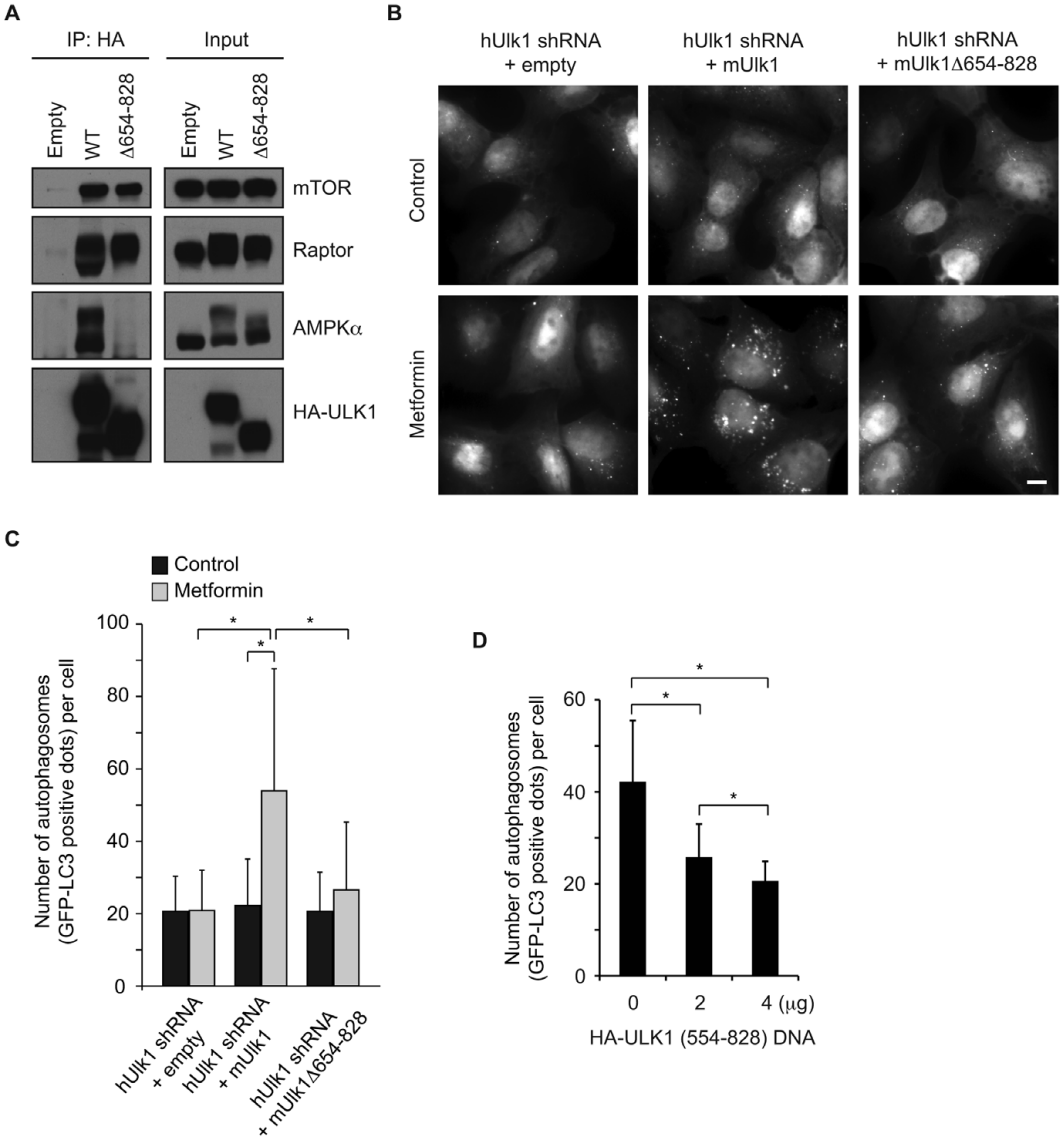
The AMPK-ULK1 interaction is important for ULK1-mediated autophagy. (A) 293T cells were transfected with empty vector or HA-tagged wild type (WT) or deletion mutant (Δ654–828) ULK1 and subjected to immunoprecipitation with anti-HA antibody followed by immunoblotting with the indicated antibodies. (B) U-2OS cells stably expressing GFP-LC3 were infected with lentivirus expressing human Ulk1 shRNA (shhUlk1) or scrambled shRNA (shScr) and subjected to selection with 1 µg/ml puromycin for 5 days. The cells were then transfected with wild type mouse Ulk1 (mUlk1), mUlk1 Δ654–828 or control empty vector for 24 h, incubated in serum-free DMEM overnight, and cultured in complete medium with or without 10 mM metformin for 20 h. The images were obtained using a fluorescence microscope. The scale bars represent 10 µm. (C) The number of GFP-LC3 dots per GFP-positive cell in (B) was quantified (mean ± s.d.; n = 81). (D) U-2OS cells stably expressing GFP-LC3 were transfected with 0, 2 or 4 µg of pcDNA3 encoding HA-tagged ULK1 deletion mutant (554–828). The total amount of plasmid DNA used for each transfection was normalized at 4 µg with empty vector. After 24 h transfection, the cells were starved in nutrient-free medium for 1.5 h, fixed in 4% paraformaldehyde, and analyzed by fluorescence microscopy. The number of GFP-LC3 dots per GFP-positive cell was quantified (mean ± s.d.; n = 100). Statistical significance was determined by Student's *t*-test and the asterisks indicate *P*<0.0001.

To determine whether the AMPK-ULK1 interaction regulates autophagy, we generated a deletion mutant ULK1 (Δ654–828) that lacks the AMPK binding domain but retains the ability to interact with mTORC1 (Fig. 3A). Transfection of mouse ULK1 into U-2OS cells expressing shRNA targeting human ULK1 restored GFP-LC3 punctate foci formation (a well-characterized marker of autophagosomes) induced by the AMPK activator metformin (Fig. 3B, C). In contrast, expression of the ULK1 (Δ654–828) mutant failed to restore metformin-induced autophagosome formation in ULK1 knockdown U-2OS cells (Fig. 3B, C). Moreover, overexpression of the AMPK-binding ULK1 fragment in U-2OS cells suppressed GFP-LC3 punctate foci formation in a dose-dependent manner after nutrient starvation (Fig. 3D). Collectively, these observations indicate that AMPK binding to ULK1 plays an important role in the induction of autophagy.

### Recruitment of 14-3-3 to the ULK1-mTORC1 complex upon AMPK activation

**Figure 4:**
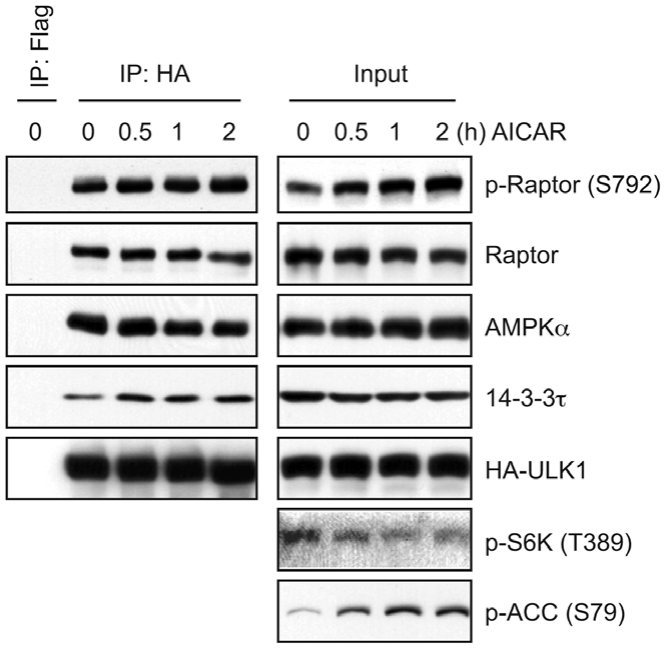
AICAR induces AMPK phosphorylation of raptor and the recruitment of 14-3-3 to the ULK1-mTORC1 complex. 293 cells stably expressing HA-ULK1 were treated with 1 mM AICAR for the indicated times and subjected to immunoprecipitation with anti-HA or control anti-FLAG antibodies. The resulting immune complexes were analyzed by immunoblotting with the indicated antibodies.

Since AMPK associates with the ULK1-mTORC1 complex and 14-3-3 binding to raptor is critical for AMPK inhibition of mTORC1 [9], we next examined whether 14-3-3 is recruited to the ULK1 complex upon AMPK activation. As shown in Fig. 4, activation of AMPK by AICAR resulted in the recruitment of 14-3-3τ to the ULK1-mTORC1 complex, which correlates with the levels of ULK1-bound Ser792 phosphorylated raptor. AICAR-induced AMPK activation and mTORC1 inactivation were determined by measuring ACC (Ser79) phosphorylation and S6K (Thr389) dephosphorylation, respectively. Although AICAR treatment modestly caused PRAS40 dephosphorylation and 14-3-3 dissociation (data not shown), it has been demonstrated that PRAS40 association is not the key event dictating the impact of raptor phosphorylation on mTORC1 kinase activity [9]. Taken together, these results suggest that activation of AMPK by energy stress recruits 14-3-3 to raptor, thereby inhibiting the mTORC1 activity in the ULK1 complex.

### AMPK phosphorylation of raptor regulates autophagy induction

**Figure 5:**
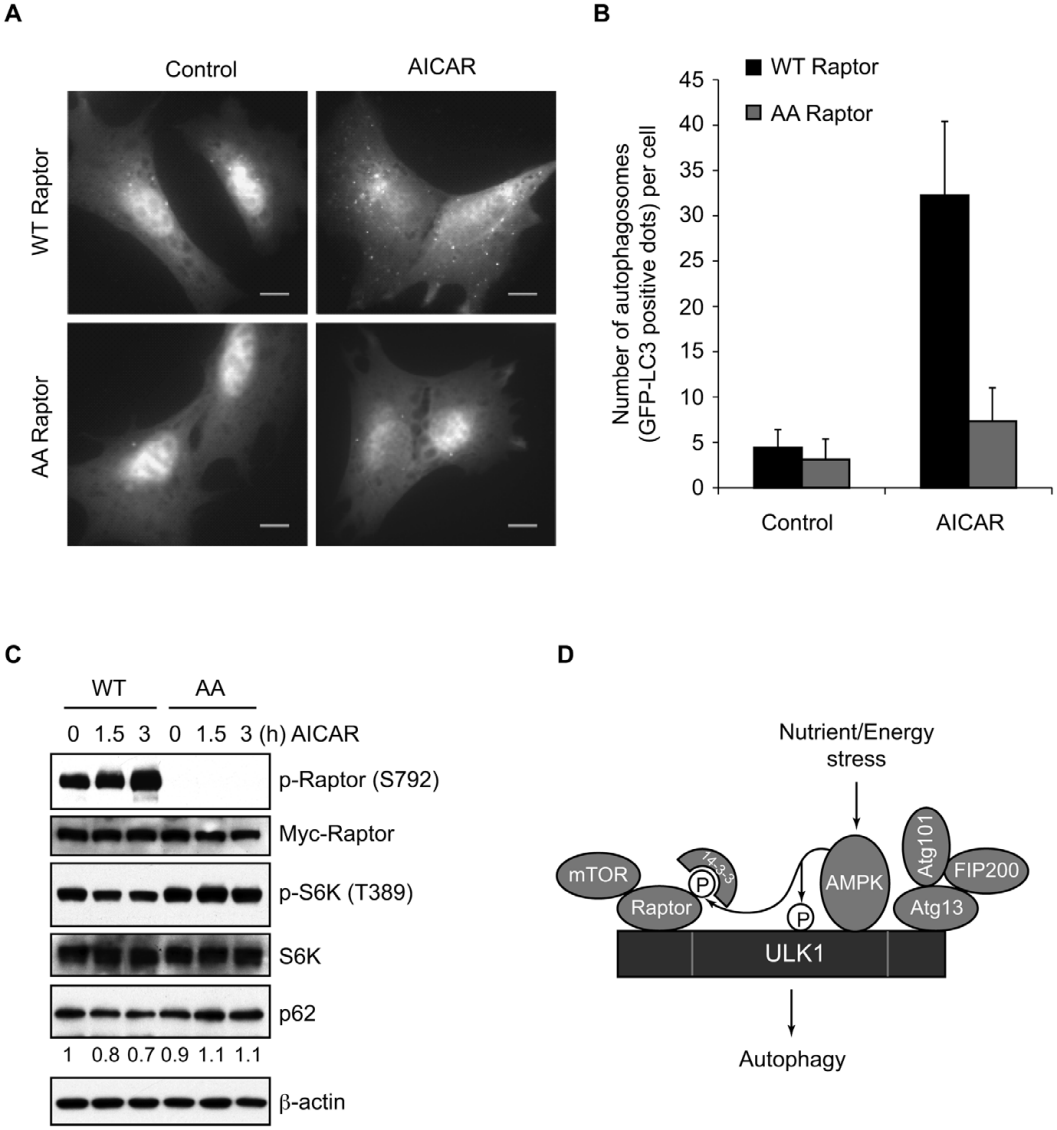
Raptor phosphorylation by AMPK is required for AICAR-induced autophagy in TSC2^−/−^ MEF cells. (A) TSC2^−/−^, p53^−/−^ MEFs stably reconstituted with wild-type raptor or S722A/S792A mutant raptor were transfected with GFP-LC3, treated with 2 mM AICAR or control vehicle for 4 h, and then analyzed by fluorescent microscopy. The number of GFP-LC3 dots per GFP-positive cell was counted (mean ± s.d.; n = 60). The scale bars represent 10 µm. (B) TSC2^−/−^, p53^−/−^ MEFs stably reconstituted with wild-type raptor or S722A/S792A mutant raptor were treated with 2 mM AICAR for the indicated times. Cell lysates were prepared in RIPA buffer and analyzed by immunoblotting with the indicated antibodies. The levels of p62 are listed relative to those of untreated WT raptor cells, which were set as 1. (C) Schematic representation of AMPK-mediated suppression of the inhibitory effect of mTORC1 on the ULK1 autophagic complex.

Although AMPK activation was shown to suppress autophagy in hepatocytes [24], it is generally agreed that AMPK is required for autophagy [25]. To determine whether the phosphorylation of raptor by AMPK is important for autophagy induction, we utilized TSC2-deficient MEF cells in which endogenous raptor was stably replaced with human wild type raptor or mutant raptor (S722A/S792A, henceforth referred to as the AA mutant) lacking AMPK phosphorylation sites as described previously [9]. The wild type raptor and AA mutant raptor cells were transfected with GFP-LC3 and treated with AICAR to activate AMPK. Autophagy was evaluated by the formation of GFP-LC3 dots. While the TSC2-deficient MEF cells with wild type raptor showed a drastic increase in the number of GFP-LC3 dots per GFP-positive cell following AICAR treatment, the cells expressing the AA mutant raptor had only a few detectable GFP-LC3 dots even after AICAR treatment (Fig 5A, B). In addition to LC3, p62/SQSTM1 is another widely used marker for autophagic flux. It has been shown that the steady state levels of p62 reflect autophagic activity [26], [27]. To examine the effect of raptor phosphorylation by AMPK on autophagic flux, we monitored p62 degradation in these cells during AICAR treatment. Immunoblot analysis revealed that autophagic flux was suppressed in TSC2-deficient cells with the AA mutant raptor (Fig. 5C). Consistent with a previous report [9], AICAR induced raptor (Ser792) phosphorylation and suppressed mTOR activity, as measured by S6K (Thr389) phosphorylation, in TSC2-deficient cells reconstituted with wild type raptor but not the AA mutant raptor (Fig. 5C). Therefore, these data suggest that AMPK-mediated phosphorylation of raptor is important for autophagy induction, presumably through activation of ULK1 by suppressing the mTORC1 inhibitory activity.

## Discussion

In this study, we have demonstrated that AMPK binds to the PS domain of ULK1, which plays a vital role in ULK1-mediated autophagy. Our findings suggest that AMPK may activate ULK1 for autophagy induction through the inhibition of mTORC1 activity by phosphorylation of raptor in the ULK1 autophagic complex (Fig. 5D). It has been reported that AMPK can suppress mTORC1 signaling through direct phosphorylation of TSC2 and raptor [7], [9]. Interestingly, FIP200, an ULK1-binding protein, has been demonstrated to interact with the TSC1/TSC2 complex through TSC1 [28]. Thus, it remains to be determined whether TSC1/TSC2 exist in the ULK1 complex and, if so, whether AMPK inhibits the mTORC1 activity not only through phosphorylation of raptor but also through phosphorylation of TSC2 in the ULK1-mTORC1 complex.

In yeast, the ability of Atg13 binding to Atg1 is regulated by its phosphorylation status [12]. While Atg13 is hyperphosphorylated in a TOR-dependent manner under nutrient-rich conditions, it is rapidly dephosphorylated under nutrient-poor conditions. The dephosphorylation of Atg13 enhances its binding to Atg1. Atg17 interacts with Atg1 through Atg13 and both Atg13 and Atg17 are required for Atg1 kinase activity, autophagy induction, and cytoplasm-to-vacuole targeting [12], [29]. In mammalian cells, however, ULK1 forms a stable protein complex with mAtg13 and FIP200 regardless of nutrient conditions [17]. Interestingly, mTORC1 dissociates from the ULK1-mAtg13-FIP200 complex under nutrient starvation conditions, and this dissociation was speculated to be the key event leading to ULK1 activation and autophagy induction [17]. Thus, it is possible that AMPK phosphorylation of raptor and subsequent recruitment of 14-3-3 to raptor cause mTORC1 dissociation from the ULK1 multiprotein complex. It is also possible that AMPK phosphorylates ULK1, resulting in a conformational change that interferes with the interaction between ULK1 and mTORC1. Alternatively, AMPK may activate ULK1 for autophagy induction through direct phosphorylation of ULK1. Indeed, purified AMPK kinase could phosphorylate recombinant ULK1 protein *in vitro* (data not shown).

The role of AMPK in autophagy regulation remains controversial. It has been shown that activation of AMPK by AICAR blocks autophagy in hepatocytes [24], [30]. In contrast, many studies suggest that pharmacological activation of AMPK by compounds, such as AICAR and metformin, induces autophagy in an AMPK-dependent manner [25], [31], [32], [33], [34]. Moreover, a recent report has demonstrated that raptor phosphorylation by AICAR-activated AMPK is required for cell cycle arrest through suppression of mTORC1 activity [9]. Consistent with these observations, our studies provide evidence that raptor phosphorylation by AMPK plays a role in autophagy induction, presumably through suppression of mTORC1 anti-autophagic activity in the ULK1 complex.

In addition, a recent study has proposed that the ULK1 complex functions in the regulation of mTORC1 activity [15]. Up-regulation of S6K1 phosphorylation at Thr389, an indicator of mTORC1 activity, was observed in mAtg13, ULK1 or ULK2 knockdown cells [15]. This result is also supported by previous findings that overexpression of Atg1 in *Drosophila* fat body has a negative effect on S6K1 phosphorylation and knockdown of ULK1 or ULK2 in mammalian cells increases S6K1 phosphorylation [35], [36]. Thus, it will be interesting to determine whether AMPK collaborates with ULK1 in the regulation of S6K1 through phosphorylation of raptor in the context of cell growth control and autophagy.

In conclusion, we have identified a new ULK1-binding protein, AMPK, which is an energy sensor coupled to cell growth control. AMPK associates with the ULK1-mTORC1 complex and promotes autophagy, at least in part, by inhibiting mTOR through phosphorylation of raptor in the ULK1 autophagic complex. Clearly, future studies are needed to further demonstrate whether AMPK also regulates mAtg13 or FIP200, such as phosphorylation, in the ULK1-mAtg13-FIP200 complex and whether ULK2 also has a role in autophagy induction mediated by AMPK. Moreover, we found that AMPK can phosphorylate ULK1 (data not shown). Therefore, further investigation of the interaction and phosphorylation regulation between ULK1 and AMPK will facilitate our knowledge on the mechanisms of the AMPK-ULK1-mTORC1 complex in autophagy induction and autophagy-associated diseases including cancer and neurodegeneration.

## Materials and Methods

### Chemical and Antibodies

Polyclonal anti-ULK1 antibody (A7481), monoclonal anti-β-actin antibody (A2228), and monoclonal anti-FLAG M2-agarose (A2220) were purchased from Sigma. Antibodies against AMPKα (2532), AMPKβ1 (4182), AMPKγ1 (4187), mTOR (2972), raptor (2280), phospho-raptor S792 (P-Raptor; 2083), p70 S6 kinase (S6K; 9202), phospho-S6K T389 (P-S6K; 9205), phospho-Acetyl-CoA carboxylase (P-ACC; 3661), and 14-3-3τ (9638) were purchased from Cell Signaling Technology. Anti-HA monoclonal antibody (HA.11; MMS-101P) and anti-c-Myc monoclonal antibody (9E10) were from Covance and EMD Biosciences, respectively. Guinea pig polyclonal anti-p62 antibody was purchased from American Research Products (03-GP62-C). Metformin (D150959) and 5-aminoimidazole-4-carboxyamide ribonucleoside (AICAR; A611700) were obtained from Sigma and Toronto Research Chemicals, respectively. Purified His6-tagged AMPKα1/β1/γ1 proteins (7439) were purchased from Cell Signaling Technology. Recombinant His6-ULK1 and His6-Bcl-XL fusion proteins were purified in *E. coli* by Ni-NTA column chromatography. Anti-His-Tag polyclonal antibody (A00174) was purchased from GenScript.

### DNA Constructions

Mouse ULK1 cDNA clones were kindly provided by Dr. Masaaki Muramatsu (Tokyo Medical and Dental University). Both full-length and fragments of mouse ULK1 cDNA were obtained by PCR amplification and subcloned into pcDNA3-HA vector. Human PRKAA2 (AMPKα2; 7262537) and PRKAB1 (AMPKβ1; 3884583) cDNA clones were obtained from Open Biosystems and subcloned into p3xFLAG-CMV10 vector (Sigma). The kinase-dead mutant of ULK1 (K46N) was made using site-directed mutagenesis kit (Stratagene). The pLKO.1-based lentiviral shRNAs targeting human ULK1 (TRCN0000000835) were purchased from Open Biosystems. The scrambled shRNA was obtained from Addgene.

### Cell Culture and Transfection

TSC2^−/−^, p53^−/−^ MEF cell lines in which endogenous murine raptor was replaced with human wild type raptor or mutant raptor (S722A/S792A) lacking AMPK phosphorylation sites [9] were kindly provided by Dr. Reuben J. Shaw (Salk Institute, La Jolla, CA). All other cell lines were purchased from ATCC. 293, 293T, MEF, and U-2OS cells were cultured in Dulbecco's Modified Eagle Medium at 37°C in 5% CO_2_. Medium was supplemented with 10% fetal bovine serum (FBS) and 1x penicillin/streptomycin. For transient expression of proteins, 293T cells were transfected with recombinant DNA plasmids using Lipofectamine 2000 (Invitrogen; 11668-019) following manufacturer's protocol. Cells were harvested 2 days after transfection for co-immunoprecipitation assay or Western blot analysis. For establishment of stable cell lines, 293 and U-2OS cells were transfected with pcDNA3-HA-ULK1 or pEGFP-LC3 plasmid, respectively, using Lipofectamine 2000 and selected by culturing in the presence of 600 µg/ml Geneticin (Invitrogen; 10131-035). For starvation treatment, cells were washed with phosphate-buffered saline (PBS) and incubated in amino acid-free DMEM without FBS (starvation medium).

### Tandem Affinity Purification of ULK1-binding Protein

The ULK1 cDNA was subcloned into the EcoRI site of pcDNA3.4-N-R1 TAP vector [37]. The amino-terminal TAP-tagged ULK1 was stably expressed in 293 cells. After starvation in Earle's balanced salt solution (EBSS) for 2 hours, the cells were lysed in cell lysis buffer (50 mM Tris-HCl, pH 8.0, 150 mM NaCl, 1% Triton X-100, 2 mM EGTA, 10 mM NaF, 1 mM Na_3_VO_4_, and protease inhibitors). The cell lysate was subjected to purification/mass spectrometry analysis as described previously [22], [37].

### Immunoprecipitation and Immunoblotting

Cell lysates were prepared in a 0.5% Triton X-100 lysis buffer (50 mM Tris-HCl, pH 7.5, 150 mM NaCl, 1 mM EDTA, 0.5% Triton X-100, 1 mM PMSF, 1 mM Na_3_VO_4_) containing a protease inhibitor cocktail (Roche; 11873580001) and phosphatase inhibitor cocktails 1/2 (Sigma; P2850/P5726). The clarified lysates were subjected to immunoprecipitation using specific antibodies in combination with protein A or G Sepharose (GE Healthcare). The resulting immunocomplexes were washed three times with lysis buffer and boiled in SDS-sample buffer. Immunoprecipitation for the mTORC1-ULK1 association was performed with a 0.3% CHAPS lysis buffer (40 mM HEPES, pH 7.4, 2 mM EDTA, 10 mM sodium-pyrophosphate, 10 mM glycerophosphate, 0.3% CHAPS, protease inhibitor cocktail, and phosphatase inhibitor cocktails 1/2) and a wash buffer (40 mM HEPES, pH 7.4, 150 mM NaCl, 2 mM EDTA, 10 mM pyrophosphate, 10 mM glycerophosphate, and 0.3% CHAPS) as described previously [38], [39]. Samples were subsequently separated by SDS-PAGE and transferred onto nitrocellulose membranes (Bio-Rad). Immunoblot analysis was performed with the indicated antibodies and visualized with Super-Signal West Pico Chemiluminescent substrate (Pierce Chemical).

### Analysis of Autophagy by Fluorescence Microscopy

TSC2^−/−^, p53^−/−^ MEF cells containing wild-type or S722A/S792A mutant raptor were transfected with GFP-LC3 plasmid using Amaxa Nucleofector NHDF kits (Lonza; F-06720) and cultured in 4-well chamber slide (Nunc; 154917). After 24 h transfection, the cells were treated with 2 mM AICAR for 4 h, fixed with 4% paraformaldehyde in PBS for 15 min, and mounted using ProLong Gold antifade reagent with DAPI (Invitrogen; P36935). Images were acquired on Olympus microscope (Olympus; IX81) coupled to the Slidebook 5.0 software.
